# Complete genome sequence of frog virus 3 isolated from a wild Eastern box turtle (*Terrapene carolina carolina*) in New Jersey, USA

**DOI:** 10.1128/mra.00112-25

**Published:** 2025-08-12

**Authors:** Amany Hassan, Joseph M. Groff, Yiyong Liu, Jo-Lynn Reno, Kathleen McMenamin-Snekvik, Preeyanan Sriwanayos, Thomas B. Waltzek

**Affiliations:** 1 Department of Veterinary Microbiology and Pathology, College of Veterinary Medicine, College of Veterinary Medicine, Washington State University 70739, Pullman, Washington, USA; 2 Department of Animal Medicine, Faculty of Veterinary Medicine, University of Alexandria 110142 , Alexandria, Egypt; 3 Office of Health and Forensics, New Jersey DEP Fish & Wildlife, Oxford, New Jersey, USA; 4 Department of Translational Medicine and Physiology, Elson S. Floyd College of Medicine, Washington State University 744660, Spokane, Washington, USA; 5 Genomics Core, Washington State University 744660, Spokane, Washington, USA; 6 Washington Animal Disease Diagnostic Laboratory, Washington State University 744660, Pullman, Washington, USA; Katholieke Universiteit Leuven, Leuven, Belgium

**Keywords:** frog virus 3, *Ranavirus rana1*, *Iridoviridae*, Eastern box turtle, *Terrapene carolina carolina*, genome

## Abstract

The complete genome sequence of a frog virus 3 (FV3) isolate from a wild Eastern box turtle was determined. Comparative genomic analyses confirmed the close genetic relationship of this virus to previous FV3 isolates from reptiles including box turtles.

## ANNOUNCEMENT

The iridovirus—frog virus 3 (FV3)—is recognized as species *Ranavirus rana1,* family *Iridoviridae* (https://ictv.global/home). FV3 exhibits low host specificity and has a global distribution with documented infections of fish, amphibians, and reptiles including common infection of North American box turtles (1, 2).

Herein is the complete genome sequence of a FV3 strain isolated from a wild Eastern box turtle that was found freshly dead in Gloucester County, New Jersey on August 7, 2023, with lesions consistent with stomatoglossitis. The turtle was refrigerated following collection prior to necropsy on August 8, 2023.

Lung, liver, kidney, and spleen were pooled and processed for virus isolation as previously described (3, 4) prior to inoculation of epithelioma papulosum cyprini (EPC) cells at 15°C. Supernatant was collected following cytopathic effect (CPE) for purification of total viral DNA using a QIAamp DNA Micro kit (Qiagen) according to the manufacturer’s instructions. A sequencing library was prepared using a DNA Prep kit (Illumina) and sequenced on a NextSeq2000 instrument (Illumina) using a P1 600-cycle reagent kit that yielded 10,143,222 paired-end reads. Data were analyzed using default options in QIAGEN CLC Genomics Workbench, version 24.0 (https://digitalinsights.qiagen.com) unless otherwise noted. Reads were trimmed, and host reads (i.e., EPC cell line) were removed by aligning the trimmed reads against a fathead minnow genome assembly (RefSeq assembly GCF_016745375.1). The resulting 3,789,496 unmapped reads were *de novo* assembled, resulting in a large contig (105,011 bp; 55% G + C). The integrity of the contig was verified by visually inspecting the alignment of the unmapped reads to the contig. Alignment of the unmapped reads to the final genome sequence incorporated 94% of reads at an average coverage depth of 4,323 reads/nt.

**Fig 1:**
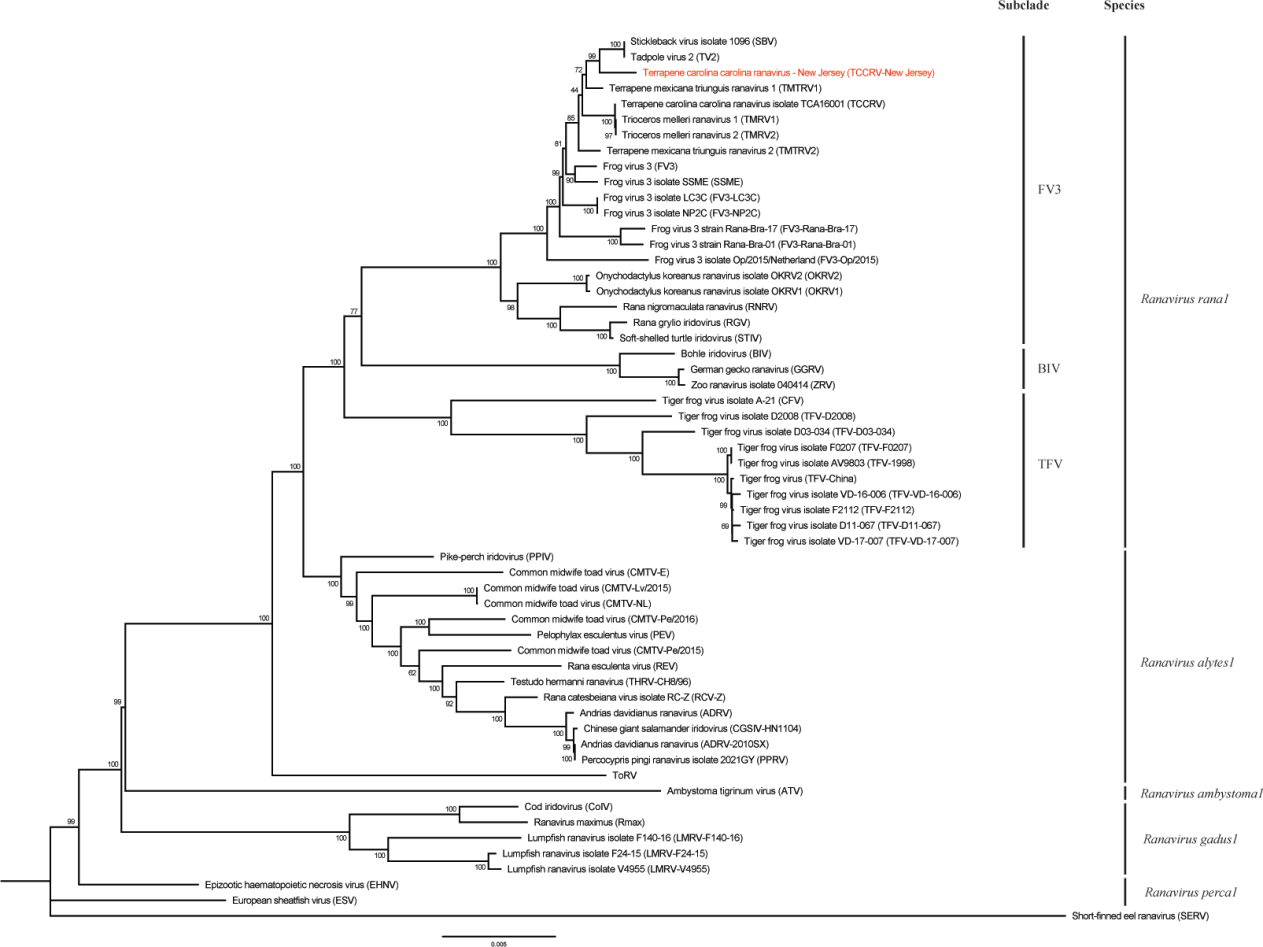
Maximum likelihood (ML) phylogram depicting the relationships of TCCRV-New Jersey (red text) to 56 ranaviruses, based on the whole genome alignments. ML analysis was performed in MEGA version 11 with default parameters and 1,000 bootstrap replicates. The bootstrap values are provided at each node. See Table 1 for the details, including abbreviations, on the viruses included in the analysis.

BLASTN analysis (Jan. 2025) of the contig revealed the top four matches (>99% identity; >96%–99% coverage) were to the complete genome sequences of FV3 strains recovered from diseased wild and managed reptiles, including box turtles (2, 5). The contig was determined to be the complete genome of the FV3 strain (terrapene carolina carolina ranavirus from New Jersey; TCCRV-NJ) by examining reads that spanned both the sequence beginning and end. Ninety-five open reading frames encoding functional proteins were predicted using the Genome Annotation Transfer Utility (https://4virology.net/virology-ca-tools/gatu/) for the TCCRV-NJ genome, with terrapene carolina carolina ranavirus (GenBank accession number MG953518) used as the reference genome. Whole genome alignments for 57 fully sequenced ranaviruses (Table 1) were generated and used in a maximum likelihood phylogenetic analysis in MEGA version 11 (6) with default parameters and 1,000 bootstrap replicates (Fig. 1). The TCCRV-NJ strain was supported as the sister group to a clade of two FV3 strains: stickleback virus (SBV) isolated from three-spined stickleback (*Gasterosteus aculeatus*) and tadpole virus 2 (TV2) isolated from northern red-legged frogs (*Rana aurora*) (7). The clade composed of the aforementioned three FV3 strains was nested within a larger FV3 clade that included strains previously reported from diseased reptiles including box turtle species (2, 5). Therefore, FV3 was confirmed in a wild Eastern box turtle from New Jersey, although the clinical significance of the virus in this case could not be definitely determined. However, the virus should be considered a pathogen of concern for wild box turtles in New Jersey.

**TABLE 1:** Virus species, virus name, abbreviations, and GenBank accession numbers of the ranaviruses used in the phylogenetic analyses

Virus species	Virus name (abbreviation)	GenBank accession number
*Ranavirus rana1*	Frog virus 3 (FV3)	AY548484
	Frog virus three isolate SSME (SSME)	KJ175144
	Trioceros melleri ranavirus 1 (TMRV1)	MG953519
	Trioceros melleri ranavirus 2 (TMRV2)	MG953520
	Terrapene carolina carolina ranavirus isolate New Jersey (TCCRV-NJ)	MG953518
	Frog virus three isolate Op/2015/Netherland (FV3-Op/2015)	MF360246
	Rana nigromaculata ranavirus (RNRV)	MG791866
	Soft-shelled turtle iridovirus (STIV)	EU627010
	Rana grylio iridovirus (RGV)	JQ654586
	Stickleback virus isolate 1096 (SBV)	JQ654586
	Tadpole virus 2 (TV2)	MZ514904
	Tiger frog virus (TFV-China)	AF389451
	Tiger frog virus isolate AV9803 (TFV-1998)	MT512504
	Tiger frog virus isolate F0207 (TFV-F0207)	MT512501
	Tiger frog virus isolate F2112 (TFV-F2112)	MT512503
	Tiger frog virus isolate D11-067 (TFV-D11-067)	MT512498
	Tiger frog virus isolate VD-16-006 (TFV-VD-16-006)	MT512499
	Tiger frog virus isolate VD-17-007 (TFV-VD-17-007)	MT512500
	Tiger frog virus isolate D03-034 (TFV-D03-034)	MT512497
	Tiger frog virus isolate D2008 (TFV-D2008)	MT512502
	Bohle iridovirus (BIV)	KX185156
	Zoo ranavirus isolate 040414 (ZRV)	MK227779
	German gecko ranavirus (GGRV)	KP266742
	Terrapene mexicana triunguis ranavirus 1 (TMTRV1)	OM963013
	Terrapene mexicana triunguis ranavirus 2 (TMTRV2)	OP852645
	Onychodactylus koreanus ranavirus isolate OKRV1 (OKRV1)	PP518040
	Onychodactylus koreanus ranavirus isolate OKRV2 (OKRV2)	PP518041
	Frog virus 3 isolate LC3C (FV3-LC3C)	PP179901
	Frog virus 3 isolate NP2C (FV3-NP2C)	PP179900
	Frog virus 3 strain Rana-Bra-01 (FV3-Rana-Bra-01)	MH351268
	Frog virus 3 strain Rana-Bra-17 (FV3-Rana-Bra-17)	MT578298
	Tiger frog virus isolate A-21 (CFV)	MW727505
	Terrapene carolina carolina ranavirus - New Jersey (TCCRV-New Jersey)	PQ844486
*Ranavirus ambystoma1*	Ambystoma tigrinum virus (ATV)	AY150217
*Ranavirus perca1*	Epizootic hematopoietic necrosis virus (EHNV)	FJ433873
	European sheatfish virus (ESV)	JQ724856
*Ranavirus alytes1*	Common midwife toad virus (CMTV-E)	JQ231222
	Common midwife toad virus (CMTV-NL)	KP056312
	Testudo hermanni ranavirus (THRV-CH8/96)	KP266741
	Tortoise ranavirus isolate 1 (ToRV1)	KP266743
	Andrias davidianus ranavirus (ADRV)	KC865735
	Andrias davidianus ranavirus (ADRV-2010SX)	KF033124
	Chinese giant salamander iridovirus (CGSIV-HN1104)	KF512820
	Common midwife toad virus (CMTV-Lv/2015)	MF004272
	Common midwife toad virus (CMTV-Pe/2015)	MF125269
	Common midwife toad virus (CMTV-Pe/2016)	MF125270
	Rana catesbeiana virus isolate RC-Z (RCV-Z)	MF187210
	Rana esculenta virus (REV)	MF538628
	Pelophylax esculentus virus (PEV)	MF538627
	Pike-perch iridovirus (PPIV)	KX574341
	Percocypris pingi ranavirus isolate 2021 GY (PPRV)	ON080858
*Ranavirus gadus1*	Lumpfish ranavirus isolate F24-15 (LMRV-F24-15)	MH665358
	Lumpfish ranavirus isolate F140-16 (LMRV-F140-16)	MH665359
	Lumpfish ranavirus isolate V4955 (LMRV-V4955)	MH665360
	Ranavirus maximus (Rmax)	KX574343
	Cod iridovirus (CoIV)	KX574342
Unclassified	Short-finned eel ranavirus (SERV)	KX353311

## Data Availability

The TCCRV-NJ genome sequence is available in GenBank under accession number PQ844486. The sequence reads are available under BioProject accession number SRR31916544.
